# Icariin Has a Synergistic Effect on the Osteoinductivity of Bone Morphogenetic Protein 2 at Ectopic Sites

**DOI:** 10.1111/os.13597

**Published:** 2023-01-10

**Authors:** Xin Zhang, Xingnan Lin, Mingjie Wang, Liquan Deng, Lingfei Wei, Yuelian Liu

**Affiliations:** ^1^ Stomatology Hospital, School of Stomatology, Zhejiang University School of Medicine, Zhejiang Provincial Clinical Research Center for Oral Diseases, Key Laboratory of Oral Biomedical Research of Zhejiang Province Cancer Center of Zhejiang University Hangzhou China; ^2^ School of Dentistry Zhejiang Chinese Medical University Hangzhou China; ^3^ Department of Oral Cell Biology, Academic Center of Dentistry (ACTA) University of Amsterdam and VU University Amsterdam The Netherlands; ^4^ School of Stomatology Zhejiang Chinese Medical University Hangzhou China; ^5^ Department of Dental Implantology Yantai Stomatological Hospital Yantai China

**Keywords:** Biomimetic calcium phosphate, Bone morphogenetic protein 2 (BMP‐2), Ectopic sites, Icariin, Osteoinductive

## Abstract

**Objective:**

Establishing biocompatible, biodegradable, osteoconductive, and osteoinductive bone materials remains a challenging subject in the research of bone healing and bone regeneration. Previously, we demonstrated the osteogenic and osteoconductive effects of biomimetic calcium phosphate (BioCaP) incorporating with Icariin and/or bone morphogenetic protein 2 (BMP‐2) at orthotopic sites.

**Methods:**

By implanting the BioCaP granules incorporated Icariin and/or BMP‐2 into the dorsal subcutaneous pockets of adult male Sprague‐Dawley (S‐D) rats (6‐7 weeks old), we investigated the osteoinductive efficacy of the samples. Micro‐computed tomography(micro‐CT) observations and histological slices were used to verify the osteoinduction of this system on the 2^nd^ and 5^th^ week. Statistical significances was evaluated using Turkey's post hoc test of one‐way analysis of variance.

**Results:**

The osteoinduction of the BioCaP incorporated with BMP‐2 or both agents was confirmed as expected. BioCaP with Icariin alone could not generate bone formation at an ectopic sites. Nevertheless, co‐administration of Icariin increased bone mineral density (BMD; *p* < 0.01) (628mg HA/cm^3^ vs 570mg HA/cm^3^) and completely changed the distribution of newly formed bone when compared with the granules with BMP‐2 alone, even though there was no significant difference in the volume of newly formed bone. In contrast, the BioCaP with both agents (37.86%) had significantly fewer remaining materials than the other groups by the end of the fifth week (53.22%, 53.62% and 48.22%) (*p* < 0.01).

**Conclusion:**

The co‐administration of Icariin and BMP‐2 increased BMD changed the distribution of newly formed bone, and reduced the amount of remaining materials. Therefore, Icariin can stimulate BMP‐2 when incorporated into BioCaP granules at ectopic sites, which makes it useful for bone tissue engineering.

## Graphical abstract

This study has demonstrated that icariin can be a stimulator of BMP‐2 when incorporated into the biomimetic calcium phosphate granules at ectopic sites.

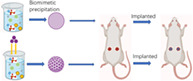

## Introduction

The regeneration of large bone defects due to assorted reasons remains a clinical challenge since it often causes incompetent healing or fractural discontinuity.^1^ This could occur due to the complicated injury environment and insufficient biological implications that initiate the bone healing cascade.^2^ Producing osteoinductive, osteoconductive, biodegradable and biocompatible bone substitute is still a challenging topic in bone healing and regeneration research. In overlapping studies of bone regenerative medicine and tissue engineering, growth factors play an indispensable role in activating endogenous repair mechanisms that can hasten functional regeneration.^3^, ^4^

The high osteoinductive ability of bone morphogenetic protein 2 (BMP‐2) has been demonstrated to induce the mesenchymal cells into osteoblasts and chondroblasts and to accelerate the recovery of open tibial fractures and spinal fusions in clinical trials.^5^ However, the rapid release and high dosage of BMP‐2 have been associated with numerous side effects, including redundant soft tissue inflammation and the abnormal ectopic bone generation. Promising biomaterials that can control of BMP‐2 release which ameliorate clinical reaction and lessen the side effects of BMP‐2, have been reported.^6^, ^7^

Icariin (ICA), a flavonol glycoside, is the major pharmacological component of Herba Epimedii, a conventional medicine herb used to treat osteo‐related illness for centuries in China. Research has indicated that ICA can facilitate osteogenic differentiation, reduce osteoclastic formation,^8^ and delivered by biological materials, and locally develop osteogenic potential.^9^

The optimal materials for substitutes, often inspired by the structure and composition of natural bone tissues, must satisfy the physicochemical characteristics required for competent bone regeneration.^10^ For this purpose, various controlled‐release bone substitutes have been applied to release agents, such as growth factors, low‐molecular‐weight drugs, to maintain the agents' long‐term activity. Most studies have focused on the therapeutic feasibility of using a single agent. The controlled release of the two agents in combination, known as dual release systems, is essential for bone tissue regeneration. Two kinds of bioactive agents, provided appropriately, will efficiently accelerate the bone regeneration of living organisms.^11^ In our previous studies, we developed a novel biomimetic calcium phosphate (BioCaP) bone substitute material that can deliver agents. To investigate the beneficial effects of the materials, a series of studies was carried out to investigate the influence of BioCaP when incorporating with agents on MC3T3‐E1 behaviors, which are a line of osteogenic precursor cells *in vitro*, and then verify the osteogenic activity of the materials at both orthotopic and ectopic sites,^8^, ^12^, ^13^, ^14^, ^15^, ^16^, ^17^, ^18^

Previously, BioCaP incorporated with ICA facilitated the osteogenic differentiation of MC3T3‐E1 cells. ICA and BMP‐2 incorporated into BioCaP materials showed superior osteogenic potential compared to BMP‐2 *in vitro*. Histological and histomorphometrical results confirmed that co‐administration of ICA into BioCaP incorporated with BMP‐2 increased bone formation in critical‐sized bone defects in the rat skulls.^8^, ^19^ As we know, the gold standard for confirming the osteoinductivity of certain biomaterial is that it can form bone tissue at ectopic sites, such as subcutaneous pockets of rats. To further demonstrate the osteoinductivity of our agents‐sustained liberating system, we applied BioCaP incorporated with ICA and/or BMP‐2 at an ectopic location in this study. Therefore, this study aimed to examine (i) whether ICA alone induced osteoinduction and (ii)whether ICA can improve BMP‐2‐induced bone formation at ectopic sites.

## Materials and Methods

### Manufacturing of BioCaP Bone Substitutes

In our previous studies, the optimal osteogenic concentrations of ICA and BMP‐2 was determined using the concentration gradient design.^8^ BioCaP bone substitutes have been manufactured by improving the classical biomimetic coating principle.^16^ In brief, a supersaturated Calcium phosphate (CaP) solution (200 mM HCl, 20 mM CaCl_2_•2H_2_0, 680 mM NaCl, and 10 mM Na_2_HPO_4_) with or without agents (ICA (10gmg/ml) or BMP‐2 (0.5mg/ml); Table 1) was buffered to a PH of 7.4 by TRIS (250 mM). Agents were added to the supersaturated CaP solution and co‐precipitated (incorporated) into the internal depot of BioCaP granules. A shaking water bath (50 agitations/min) was used to incubate the solution at 37°C for 24 h. Rapid precipitation occurred when the PH reached approximately 6.25. Precipitation was retrieved after 24 h of incubation, gently rinsed with distilled water, filtered through filter paper, and compressed into tablets with a diameter of 5 mm and thickness of 0.4 mm, using a vacuum exhaust filtering method with a vacuum filter (0.22‐μm pore, Corning, NY, USA) and an air pump. After drying at room temperature, BioCaP tablets were ground and filtered to obtain materials with a size of 0.3–0.6mm using porous metallic mesh filters. All experimental procedures were performed under strict aseptic conditions.

**TABLE 1:** Concentrations of agents added to the supersaturated CaP solutions before buffering

	Concentration of agents, mg/L
BioCaP+ICA	10
BioCaP+BMP‐2	0.5
BioCaP+ICA + BMP‐2	10 + 0.5

### In vivo Experiments

#### Experimental Animal Model and Grouping

Adult male standard deviation (S‐D) rats (6–7 weeks‐old, 200–220g body weight) were used in this study. Six rats were assigned to each group, and a total of 24 rats were used. Three experimental groups and one control group were analyzed. This study  was approved by Ethical Committee of School of Stomatology, Zhejiang Chinese Medical University (IRB/IEC number: ZSLL‐2014‐47).

The groups inclued the

1. BioCaP granules (BioCaP, negative control)
2. BioCaP granules incorporated with ICA (BioCaP+ICA, experimental);
3. BioCaP granules incorporated with BMP‐2 (BioCaP+BMP‐2, positive control); and
4. BioCaP granules incorporated with ICA and BMP‐2(BioCaP+ICA + BMP‐2, experimental)

#### Surgery Procedure and Histology

**Fig. 1:**
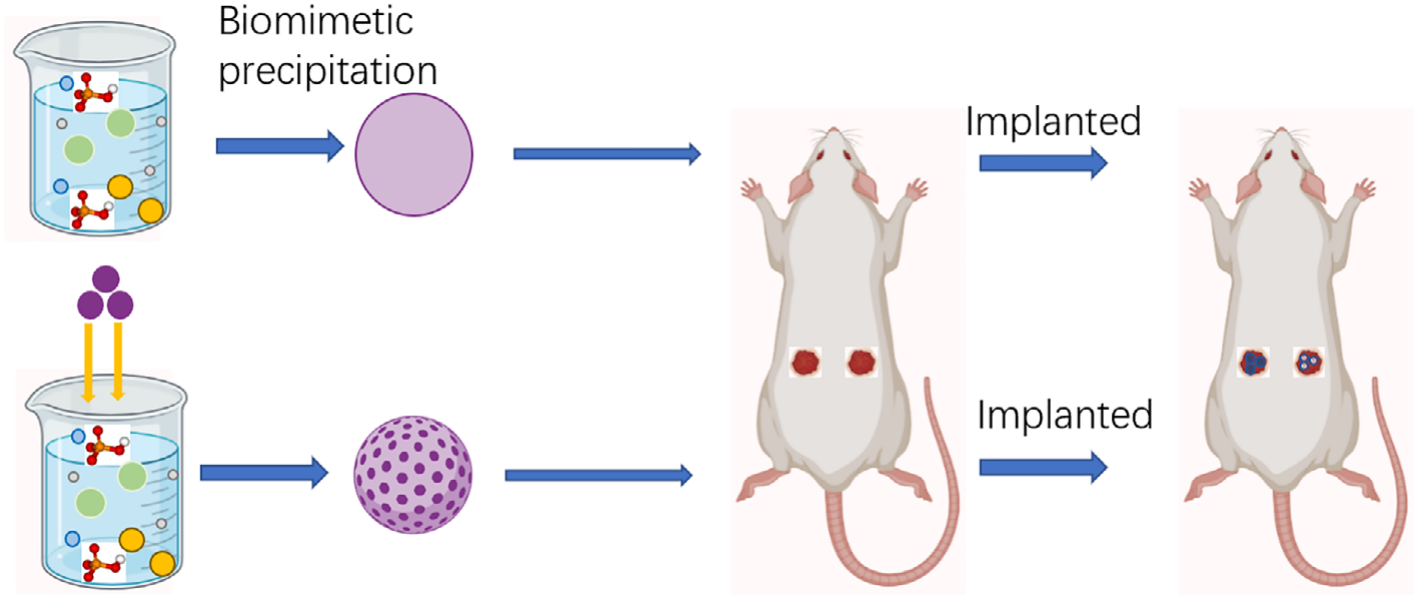
The process of BioCaP biomimetically incorporated with agents and *in vivo* study with the ectopic bone models in rats.

Twenty‐four adult male S‐D rats which (6–7 weeks old)weighing 200‐220g were used in this study. Male rats were used to avoid any differential effects, considering the sex of the animals. The surgery was performed under general anesthesia using 1% pentobarbital (0.1 mL/100 g). The dorsal of rats was shaved and disinfected for aseptic surgery. Two 1 cm skin incisions were created on the back and subcutanous tissue, one on the left and one on the right, and bluntly separated laterally. The samples were distributed to the S‐D rats using a systematic random sampling protocol.^20^ The rats were numbered from 1 to 24 and each rat was implanted with one of two groups of BioCaP materials. BioCaP granules, each weighing 0.2 g, were implanted into the dorsal subcutaneous pockets of each S‐D rat, one on the left and one on the right. The samples were fixed by suturing the incision (Fig. 1).

By the end of the 2nd and 5th weeks, the S‐D Rats (n = 6 rats per group) were euthanized separately. The retrieved materials with tissues 3–5 mm from the defect were chemically fixed, dehydrated with conventional alcohol, and embedded in an methylmethacrylate block.^16^ The samples were cut using a hard tissue microtome vertically to the long axis, into 10–12 slices for each sample. Each slice was 600 μm thick, and 1 mm apart. All slices were mounted on square polyetherimide holders and polished until no obvious scratches were observed on the surface. The slices were surface‐stained with McNeal's Tetrachrome, toluidine Blue and basic Fuchsine,^21^, ^22^ for the histomorphometric analysis of a various parameters using a Nikon‐Eclipse light microscope.

#### Micro‐Computed Tomography (Micro‐CT) Analysis

Micro‐CT was performed using a Siemens Inveon CT scanner (Siemens Medical Solutions, Knoxville, TN, USA). The parameters for histomorphometric analysis were as follows: 80 kV voltage, 500 μA current, 1500ms exposure time, 360° rotation, 360 projections and effective pixel size 9.29 μm. Acquisitions were collected using built‐in machine software and reconstructed using a filtered back projection algorithm.

#### Histomorphometric Analysis

After digitally recording a subjective histological observation, an objective quantitative histomorphometric analysis was conducted using 10 slices of each sample. Microscope images were recoded under a light microscope. The remaining graft materials and newly formed bone were quantified using an image analysis system (Image Pro Express, USA).

#### Statistical Analysis

All quantified data are presented as means ± standard deviations (SDs). Statistical significance was evaluated using a Tukey's *post‐hoc* test of one way analysis of variance Using the analysis software SPSS 16.0 (SPSS Science). Statistically significant was set at *P* < 0.05.

## Results

### Micro‐CT Analysis

**Fig. 2:**
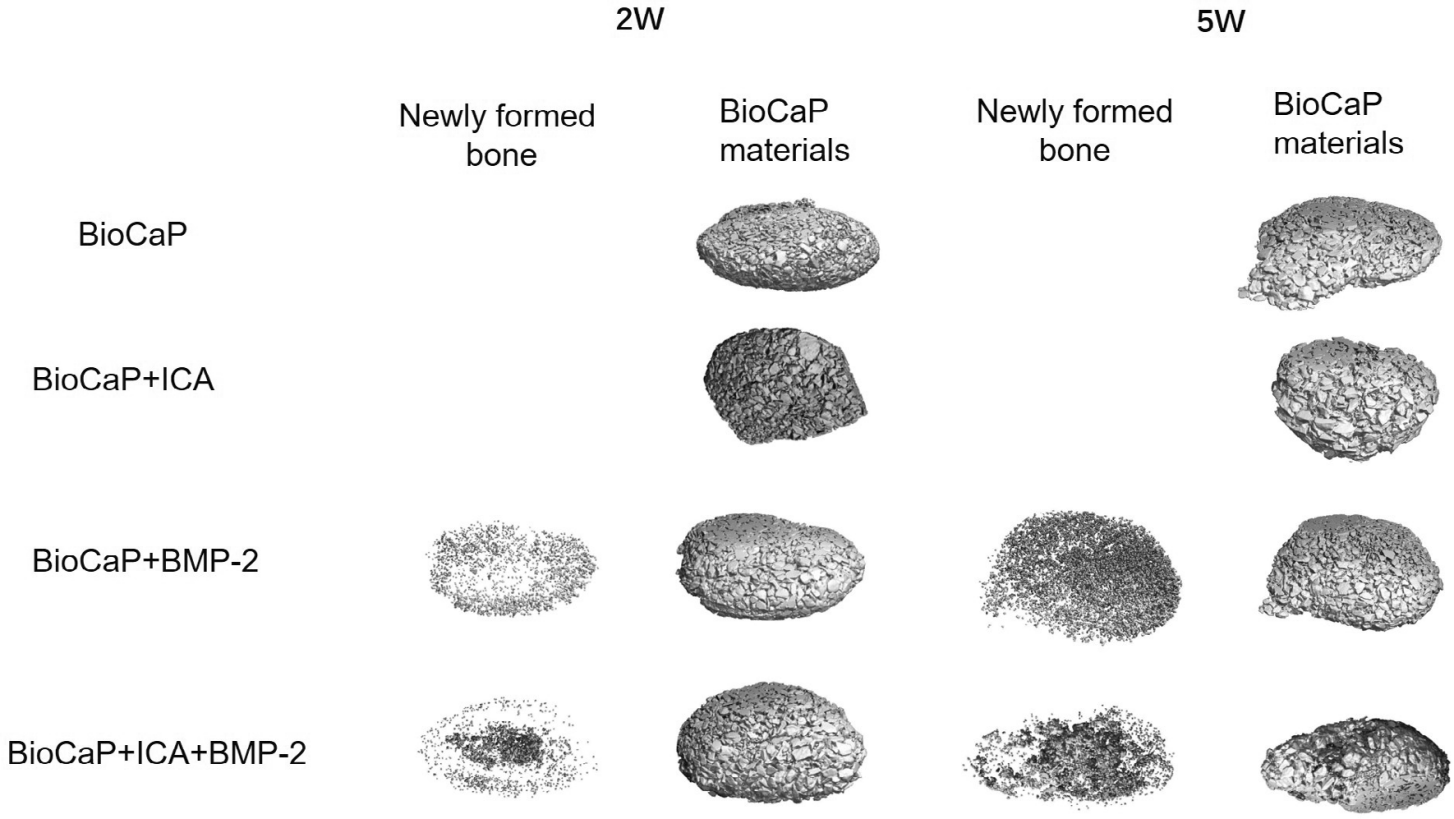
The Micro‐CT images presenting the newly formed bone and BioCaP materials in the rats' dorsal subcutaneous pockets 2 and 5 weeks after implantation with BioCaP, BioCaP+ICA, BioCaP+BMP‐2 and BioCaP+ICA + BMP‐2.

Micro‐CT images showed that BioCaP alone and granules incorporated with ICA showed no new bone formation. Interestingly, bone appeared to be gathered in the center of the BioCaP samples incorporated with both agents. However, the newly formed bone was uniformly distributed throughout the entire BioCaP sample incorporated with BMP‐2 (Fig. 2).

**Fig. 3:**
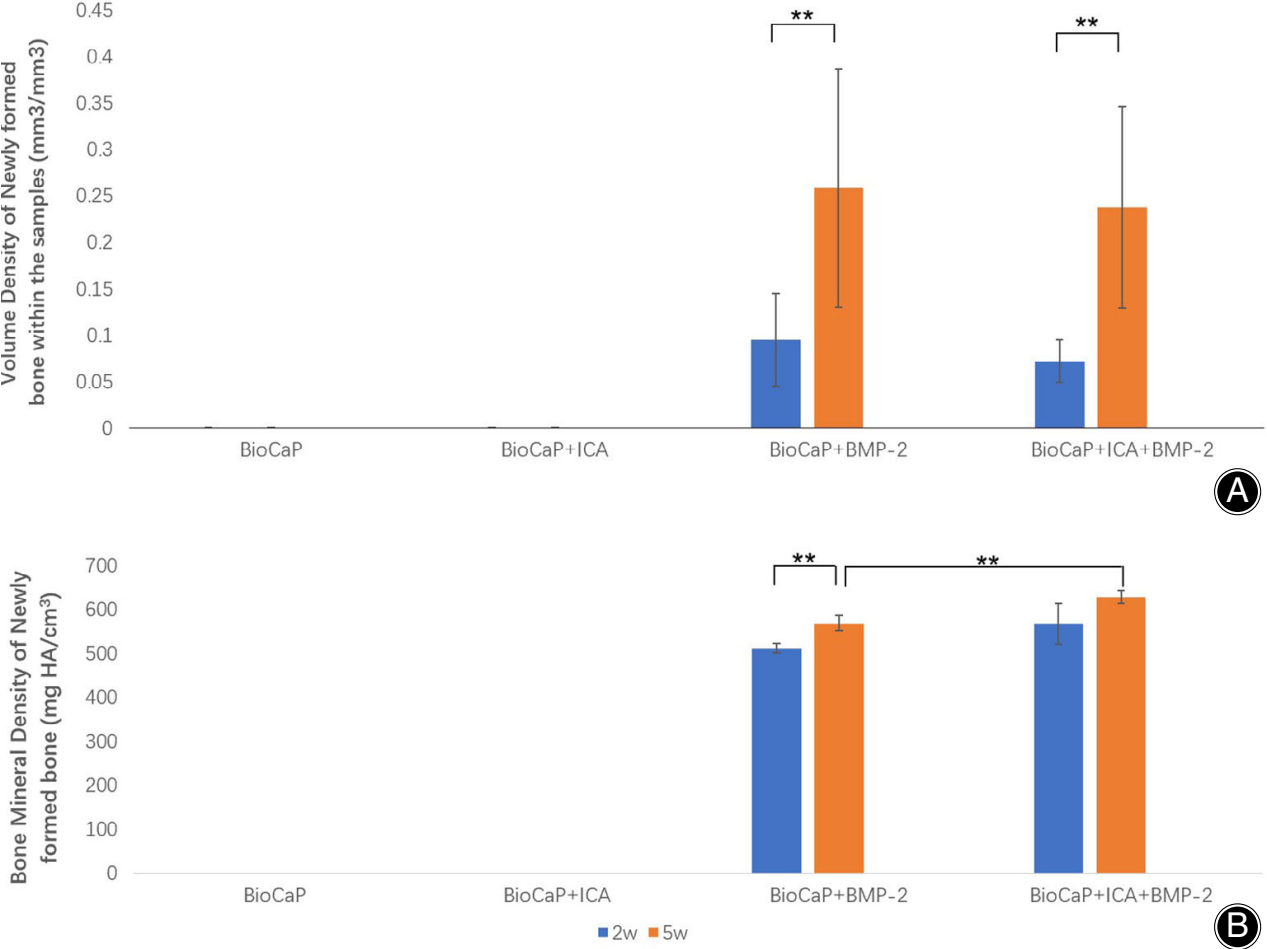
The analyzed Micro‐CT data. (A) The volume of newly formed bone in the samples. (B) The bone mineral density (mg HA/cm^3^). The slices' mean value (n = 6) is presented along with the SD. **P < 0.01.

The difference between the newly formed bone were calculated (Fig. 3). Micro‐CT results indicated no differences in the volume density of newly formed bone (bone volume/total volume [mm^3^/mm^3^]) between the BioCaP group with BMP‐2 and the BioCaP group with incorporated ICA and BMP‐2 by the end of the 2^nd^ and 5^th^ weeks. After 5 weeks, the newly formed bone was increased on both BioCaP incorporated with BMP‐2 and BioCaP with the two agents compared with 2 weeks of implantation. After 5 weeks, the addition of ICA (628 mg HA/cm^3^) significantly increased the bone mineral density (BMD; mg HA/cm^3^) of BioCaP incorporated with BMP‐2 (570 mg HA/cm^3^, while there were no differences at 2 weeks.

### Descriptive Light Microscopy

**Fig. 4:**
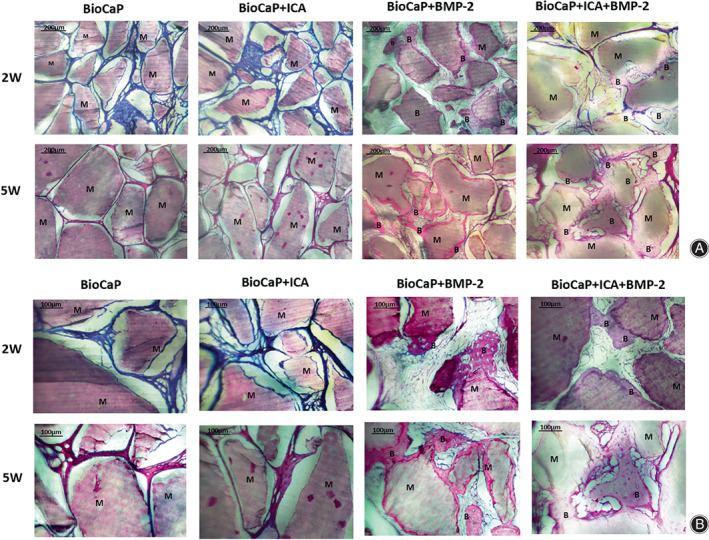
The histological micrographs by the end of the 2^nd^ and 5^th^ weeks posteoperatively. (A) Scale bar=200um. (B) Scale bar=10 um. The BioCaP material (M) was surround by newly formed bone (B) in the BioCaP+BMP‐2 and BioCaP+ICA + BMP‐2 groups. There was no new bone formation in the BioCaP and BioCaP+ICA groups.

For 2 or 5 weeks, no bone could be observed in the groups of BioCaP granules alone and BioCaP with ICA. Newly formed bone was observed in contact closely around the BioCaP granules interiorly incorporated with BMP‐2. The granules with BMP‐2 were encapsulated in a layer of new bone. The layer of new bone between the granules can be interconnected. Interestingly, although there was newly formed bone in the BioCaP with both agents, it was not significantly larger than the BioCaP with BMP‐2 alone, it can independently exist not just around the materials (Fig. 4).

Descripitive Light microscopy At 2 or 5 weeks, no bone was observed in the BioCaP granules alone or BioCaP with ICA groups. Newly formed bone was observed in close contact with the BioCaP granules interiorly incorporated with BMP‐2. Granules containing BMP‐2 were encapsulated in a layer of new bone. The layer of new bone between the granules can be interconnected. Interestingly, although the newly formed bone in BioCaP with both agents was not significantly larger than that in BioCaP with BMP‐2 alone, it can independently exist, not just around the implants (Fig. 4).

### Histomorphometric Results

**Fig. 5:**
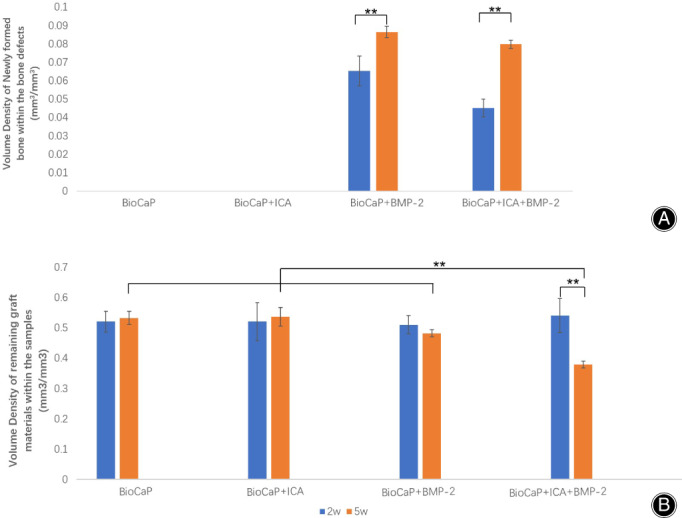
(A) The volume of newly formed bone in the samples and (B) remaining graft materials within the samples after 2 and 5 weeks postoperatively for each group. The slices' mean value (n = 6) per group is presented along with SD. **P < 0.01.

Quantitative evaluation of new bone formation at 2 and 5 weeks after surgery is shown in Fig. 5A. No new bone formation was observed in the BioCaP granules alone or in BioCaP incorporated with ICA. By the end of the 5^th^ week, both BioCaP with BMP‐2 (8.64%) and BioCaP groups with both agents (7.98%) produced a significantly higher volume density of newly formed bone than after 2 weeks (6.52% and 4.51%). However, after 2 and 5 weeks, there was no significant differences between the BioCaP with BMP‐2 and BioCaP with either agents (Fig. 5A).

There were no statistically significant differences in the remaining graft materials between the four groups after 2 weeks. Nevertheless after 5 weeks, the BioCaP with both agents (37.86%) had significantly fewer remaining materials than the other three groups (53.22%, 53.62% and 48.22%) (Fig. 5B).

## Discussion

The most commonly transplanted solid tissue is bone,^23^ and bone grafting is indispensable for reconstructing bone defects. The selection of preferable graft materials depends on the volume needed for bone defects, newly formed bone quality, accessibility, and cost. The best properties of osteoconductivity, osteoinduction, and osseo‐integration are the basic characteristic for ideal available bone graft materials.^24^ Bone graft substitutes should enable vascular invasion and cellular and bioactive factor infiltration. Mesenchymal stem cells (MSCs) are then recruited and stimulated to differentiate by the osteoinductive factors.^25^

The adsorption of BMP‐2 delivered by bone substitutes is usually related to a high dose burst release and undesirable osteoinduction. Some studies have reported that the chemical modification of biomaterials which can control the release of BMP‐2 may also. decrease the bioactivity of BMPs.^6^, ^26^

In our previous study, two types of bioactive molecules (ICA and BMP‐2) were used to improve the osteogenic effect of bone graft materials. The osteogenic effects of BioCaP incorporated with ICA and/or BMP‐2 *in vitro* and in periosteal osteogenesis have been established.^8^, ^12^, ^13^, ^14^, ^15^, ^16^, ^17^, ^18^ To further demonstrate the osteoinductivity of our agent‐sustained liberating system, we applied BioCaP incorporated with ICA and/or BMP‐2 at ectopic locations in this study.

Osteoconduction is defined as the capability of the biomaterials to offer a microscopic bio‐derived bone scaffold upon which bone ingrowth can occur. Without the osteoconductive scaffold offered by these materials, *de novo* new bone formation can be inhibited, causing suboptimal materials incorporation. Osteoinduction describes the capability of materials to recruit MSCs and stimulate them to differentiate into osteoblasts and chondroblasts. This process depends on a various growth and osteoinductive factors, such as BMPs, which induce MSCs to differentiate into osteoprogenitors.^27^ The gold standard for osteoinductivity of certain biomaterial is the formsation of bone tissue at ectopic sites, such as the subcutaneous pockets of rats.^16^

### BMP‐2 Had an Osteoinductive Effect at an Ectopic Site

The slow and lasting release of BMP‐2 facilitates the osteoinductivity of BMP‐2.^28^ In our study, new bone formed in the BioCaP samples of incorporated with BMP‐2 was wrapped around the granules. In other words, the BioCaP materials were encapsulated by the newly formed bone, which may support the good biocompatibility, osteoconduction and osteoinduction of these scaffolds. This was in line with the findings from our study that BioCaP granules delivering BMP‐2 make the materials efficiently produce osteoinductive compounds *in vivo*.^16^ Moreover, this new format of BMP‐2 incorporated BioCaP granules also showed a sustained release of agents of this system, while there were significant differences in bone volume between the 2‐ and 5‐weeks samples.

### ICA Itself Had no Osteoinductive Effect at the Ectopic Sites

Our previous study demonstrated that BioCaP incorporated with ICA could significantly enhance the osteogenic differentiation of MC3T3‐E1 cells but had no influence on cell proliferation. BioCaP with ICA significantly facilitates the bone formation in critical sized calvarial defects in S‐D rats.^8^

The BioCaP granules group with BMP‐2 alone induced new bone formation at the ectopic site, as expected in this study, which is consistent with our previous study.^16^ However, the BioCaP granules incorporated with ICA alone showed no bone formation, indicating that ICA had low osteoinductivity.

Our findings strongly suggested that ICA alone has osteoconductive characteristics but not osteoinductive characteristics.

### Adding ICA Did Not Increase the Bone Volume of BioCaP Incorporated with BMP‐2, but it Had Higher BMD and Changed the Distribution of Newly Formed Bone

Although adding ICA did not increase the total bone volume, BMD significantly improved after 5 weeks. “Xian Ling Guo Bao” is a multicomponent formulation of Chinese medicine with ICA as the main ingredient. In a 24‐month randomized, double‐blind, and placebo‐controlled test, treatment with the traditional dose of “Xian Ling Guo Bao” indicated the safe and a statistically significant facilitation of BMD of the lumbar spine in postmenopausal women.^29^ ICA can reverse low BMD in older caged laying hens.^30^ Supplying ICA significantly increased femur and tibia BMD in older caged laying hens, similar to the efficiently osteogenic action of ICA on ovariectomy‐induced osteoporosis in rats and D‐galactose‐induced osteoporosis in mice.^31^, ^32^

Although the adding ICA did not promote the bone formation of BioCaP incorporated with BMP‐2, the newly formed bone was observed both around and independently from the granules, whereas the new bone formed only encapsulated the BioCaP granules with BMP‐2 alone. Moreover, the distribution of bone was completely distinct when ICA was added to the BioCaP with BMP‐2. Interestingly, although we did not accurately quantify it, in the micro‐CT observation and further histological slices, the new bone formed in the group of both agents was mostly in the central region of the implanted area, while the bone formed in the granules with BMP‐2 alone was distributed evenly. In other words, in the BioCaP group incorporating both ICA and BMP‐2, more bone seems to have formed in the center of the sample than in the periphery.

As we know, the capability to differentiate into osteoblasts of MSCs makes them the prerequisite for ectopic osteogenesis.^33^ MSCstend to differentiate into an osteoblasts lineage and a pro‐angiogenic secretome, which stimulates *in vitro* prevascularization or promotes *in vivo* neovascularization.^34^ As we know the injured tissues may present particular messenger signals that result in infiltration, trafficking and homing MSCs to injury sites.^25^ In the early stage of ectopic bone formation, MSCs enter the sample sites via the blood supply. Unidirectional collagen fibers were observed, and spindle‐shaped osteoblasts were recruited.^35^

BMP‐2 can effectively enhance vascular endothelial formation within 48 h prior to the growth of cartilage, which further facilitates a better blood supply.^36^ ICA and BMP‐2 delivered by BioCaP materials are released slowly and last, thus inducing vascular endothelial appearance, while a blood supply is a prerequisite for bone formation. Meanwhile, ICA may develop its protective effects by reducing the destruction of the extracellular matrix and regulating MSCs' osteogenic differentiation through the mitogen‐activated protein kinase signaling pathway.^37^ We hypothesized that the addition of ICA may increase the release of BMP‐2 in the center rather than in the periphery through an unknown mechanism. Thus, a higher vascularity is expected in the center, with higher amounts of MSCs, which results in higher local bone formation.

The osteogenic effect of ICA in the bone environment has been confirmed by numerous studies, while few studies have verified its osteoinductive effect at ectopic sites. Here, we show for the first time that ICA and BMP‐2 can be delivered and released properly and still have good biocompatibility and osteoinductivity.

Our data showed that ICA alone had little osteoinduction and failure to stimulate bone formation when released by the scaffold in the dorsal subcutaneous pockets of rats. Interestingly, when BMP‐2 was added with ICA incorporated into the BioCaP granules, the form of bone distribution and the bone mineral density became widely different. Moreover, this new format of BMP‐2 or ICA‐BMP BioCaP granules also showed a sustained release of agents of this system, while there are significant differences in bone volume between the 2‐ and 5‐week samples.

### The BioCaP with Both Agents Had Less Remaining Graft Materials than Other Three Groups after 5 Weeks

By the end of the 5^th^ week, the BioCaP with both agents had fewer remaining graft materials than the other three groups, with no differences after the 2^nd^ week. Fewer remaining graft materials result in faster degradation of the materials. The degradability of a CaP‐based graft is crucial for the efficacy and longevity of its biological efficiency.^38^ During the 1^st^ week of the post‐surgical phase, multinucleated foreign body giant cells (FBGC) are recruited to the implantation site prepared against foreign grafts as part of the inflammatory response and embark on their destructive mission by attacking the biomaterials. These agents can be released when degrading the inorganic matrix to further facilitate osteogenic efficiency. FBGCs have a significantly greater resorption efficiency and play a fundamental role in the degradation of biomaterials.^39^

Osteoclasts participated in the degradation of CaP‐based biomaterial following exclusive domination by FBGCs during the initial phase. Normally, the rapid degradation can be seen during and after the first 3 weeks, which led to a significant decrease in the remaining graft biomaterials by the end of the 5^th^ week.^40^ This may explain the reduction in the remaining materials after 5 weeks in this study.

The degradation of materials also represents the liberating of agents incorporated into BioCaP. In this study, BioCaP with both agents had fewer remaining graft materials than the other three groups by the end of the 5^th^ week, while the newly formed bone showed no differences between BioCaP with BMP‐2 and BioCaP with both agents, possibly due to the limited amount of degradation.

### Stengths and Limiatations

The strengths of this study are that it examined the osteoinductive efficiency of ICA alone and with the co‐administration with BMP‐2 at ectopic sites. We have shown for the first time that ICA and BMP‐2 can be incorporated, delivered and released properly and simultaneously have good biocompatibility and osteoinductivity. Moreover, this new format of BMP‐2 or ICA ‐BMP BioCaP granules also showed a sustained release of agents of this system, while there were significant differences in bone volume between 2‐ and 5‐weeksamples. A limitation of this study was that only one concentration of ICA and BMP‐2 was selected for the supersaturated CaP solution when manufacturing the BioCaP bone substitute. Additional measurements, such as lamellar bone and cartilage formation, which can indicate osteogenic and osteoinductive activities, should be included in further studies.

### Conclusions

In the present study, we applied our slow‐release bone tissue engineering drug delivery system at ectopic sites. BioCaP with ICA alone did not generate ectopic bone formation. We indicated osteoinduction of the BMP‐2 or ICA + BMP‐2 BioCaP scaffolds using an ectopic model, which is the “gold standard” as evidence of osteoinductive activity. However, co‐administration of ICA and BMP‐2 did not increase bone volume but resulted in better bone mineral density and changed the distribution of newly formed bone in the samples.

## Author Contributions

All authors participated in this study and fully approved to submit this manuscript. Conceptualization, XZ and YLL; Methodology, XZ and YLL; Investigation, XNL, MJW, LQD and LFW; Formal Analysis, XZ and XNL; Resources, MJW, LQD and LFW; Writing, Reviewing and Editing, XZ, XNL, LFW and YLL; Supervision, YLL; Funding Acquisition, XZ and YLL.

## Funding Information

This study was supported by The Zhejiang Provincial Natural Science Foundation of China (LQ19H280008) and Taishan Scholar funding to Dr. Yuelian Liu, Shandong, China.

## CONFLICT OF INTEREST

The authors declare no conflict of interest.
